# Recent advances in the development of RIPK2 modulators for the treatment of inflammatory diseases

**DOI:** 10.3389/fphar.2023.1127722

**Published:** 2023-03-07

**Authors:** Anh-Tuan Pham, Amanda Franceschini Ghilardi, Lijun Sun

**Affiliations:** Center for Drug Discovery and Translational Research, Beth Israel Deaconess Medical Center, Harvard Medical School, Boston, MA, United States

**Keywords:** RIPK2, SBDD, kinase inhibitors, PROTACS, PK/PD, inflammatory diseases

## Abstract

Receptor-interacting serine/threonine kinase 2 (RIPK2) is a vital immunomodulator that plays critical roles in nucleotide-binding oligomerization domain 1 (NOD1), NOD2, and Toll-like receptors (TLRs) signaling. Stimulated NOD1 and NOD2 interact with RIPK2 and lead to the activation of nuclear factor kappa B (NF-κB) and mitogen-activated protein kinases (MAPK), followed by the production of pro-inflammatory cytokines such as TNF-α, IL-6, and IL-12/23. Defects in NOD/RIPK2 signaling are associated with numerous inflammatory diseases, including asthma, sarcoidosis, inflammatory bowel disease (Crohn’s disease and ulcerative colitis), multiple sclerosis, and Blau syndrome. As RIPK2 is a crucial element of innate immunity, small molecules regulating RIPK2 functions are attractive to establish novel immunotherapies. The increased interest in developing RIPK2 inhibitors has led to the clinical investigations of novel drug candidates. In this review, we attempt to summarize recent advances in the development of RIPK2 inhibitors and degraders.

## Introduction

Receptor-interacting serine/threonine kinase 2 (RIPK2) is a dual specific kinase downstream of the nucleotide-binding oligomerization domain 1 (NOD1) and NOD2 signaling pathways, which play essential roles in regulating the innate immunity (Irving et al., 2014). RIPK2 also plays a role in adaptive immunity as it is involved in CD4^+^ T cell proliferation and T-helper cell development (Chin et al., 2002). Dysregulation of NOD/RIPK2 signaling is implicated in the development of several inflammatory diseases, including sarcoidosis, Blau syndrome, Crohn’s disease (CD), and ulcerative colitis (UC) (Hugot et al., 2001; Lesage et al., 2002; Hysi et al., 2005; McGovern et al., 2005; Tanabe et al., 2006; Stronati et al., 2008; Negroni et al., 2009; Stronati et al., 2010; Rose et al., 2011; Strober et al., 2014; Honjo et al., 2021). Therefore, modulation of the NODs/RIPK2 signaling pathway is a promising approach for the development of novel immunotherapies. Exquisite drug discovery programs have resulted in highly potent RIPK2 inhibitors, and some have entered human clinical trials. This review aims to summarize the recent progress in the designs and characterizations of RIPK2 inhibitors and degraders from a medicinal chemistry perspective. Details of the NODs/RIPK2 cellular signaling as well as their roles in inflammatory diseases can be found in recently published reviews (Chen et al., 2009; Jun et al., 2013; Caruso et al., 2014; Irving et al., 2014; Humphries et al., 2015; Ermine et al., 2022).

## RIPK2 structure and regulation

RIPK2 (also known as RIP2, RICK, CARD3, or CARDIAK) is a 541 aa-protein belonging to the receptor-interacting protein (RIP) kinase family. It was first described independently in 1988 by Thome et al. (1998); Inohara et al. (1998); McCarthy et al. (1998). Similar to other members of the RIP kinase family, RIPK2 contains a characteristic homologous kinase domain (KD) at the N-terminal region (aa 1-310). This KD is linked to a caspase-recruiting domain (CARD) (aa 454-541) by a bridging intermediate domain (INTD). Most structural studies have focused on the RIPK2 KD because the functions of KD are independent of the CARD, and kinase inhibitors are well-established therapeutic agents in cancers and inflammatory diseases (Ferguson and Gray, 2018; Cohen et al., 2021; Ayala-Aguilera et al., 2022; Lee et al., 2022).

**FIGURE 1:**
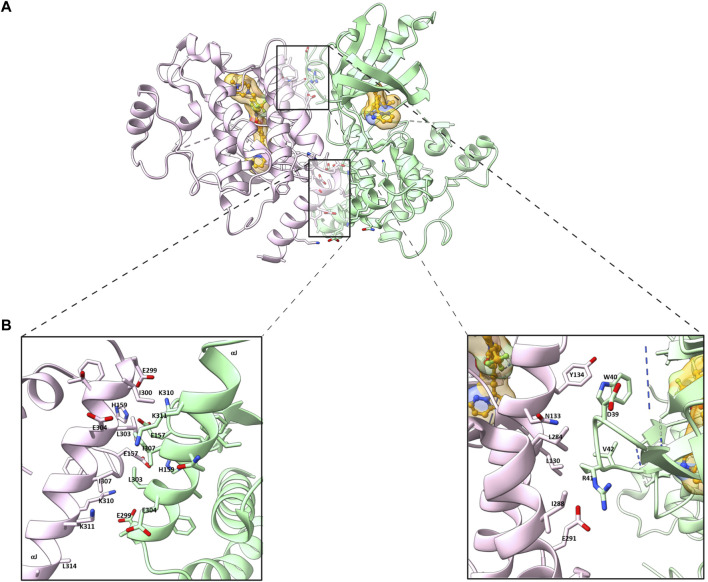
**(A)** Homodimeric packing of RIPK2 (PDB ID: 4C8B) **(B)** Details in the interface between two RIPK2 monomers. Monomers are shown in green and pink, respectively. The ligand is shown as golden (space-filled).

Crystallographic studies of ligand bound and apo (ligand-free) RIPK2 KD have provided insightful three dimensional (3D) structural information (Canning et al., 2015; Charnley et al., 2015; Pellegrini et al., 2017). The ligand (ponatinib) bound RIPK2 crystal structure reveals a homodimeric packing arrangement with a highly symmetrical protein interface (Figure 1A). The αJ helices bind to each other in an antiparallel fashion by hydrophobic effects and H bonding of Lys310 and Glu299 to His159 and Glu157 (Figure 1B). Additional interactions are present between the β2-β3 loop of one subunit and the αE and αI helices of the other. In analogy with ligand-bound crystal structure, two molecules of RIPK2 form a dimer in the apo-structure, and the two monomers are held together by twenty hydrogen bonds and fourteen salt bridges (Charnley et al., 2015). A more detailed analysis of RIPK2 crystal structure in both active and inactive states was described by Pellegrini et al. (Pellegrini et al., 2017). In the active form, RIPK2 also appears as a 2-fold symmetric dimer with the N-termini forms antiparallel *ß*-strands that are further stabilized by His71 and Arg74. Disruption of this interaction in the mutants RIPK2^R74A^, RIPK2^R74D^, or RIPK2^R74H^ dramatically decreases kinase activation and protein stability *in vitro*.

The auto-phosphorylation at the activation segment (AS) on Ser174, Ser176, Ser178, and Ser180 or Ser181 stabilizes RIPK2 activation by inducing conformational changes in the AS, from a helix to a disordered loop with the first phosphorylation event happens in trans between kinase dimers. The structure of the Lys209 loop is also involved in the activation of RIPK2. Particularly, Lys209, at the end of the Lys209 loop (residues 200-210), forms H bonds with the carbonyl-oxygens of Pro277 and Arg280 and stabilizes a helical turn conformation of residues 277-280. The rest of the Lys209 loop is disordered in the active form. On the contrary, in the inactive form, the Lys209 loop is structured and contains a short helix. Since the Lys209 loop is in a dynamic equilibrium between a helical or disordered conformation, the AS can move in kinase activation. Thus, the proximity of the phosphorylated AS can alter the conformation of the Lys209 loop, followed by changing the accessibility of Lys209 to ubiquitination machinery.

**FIGURE 2:**
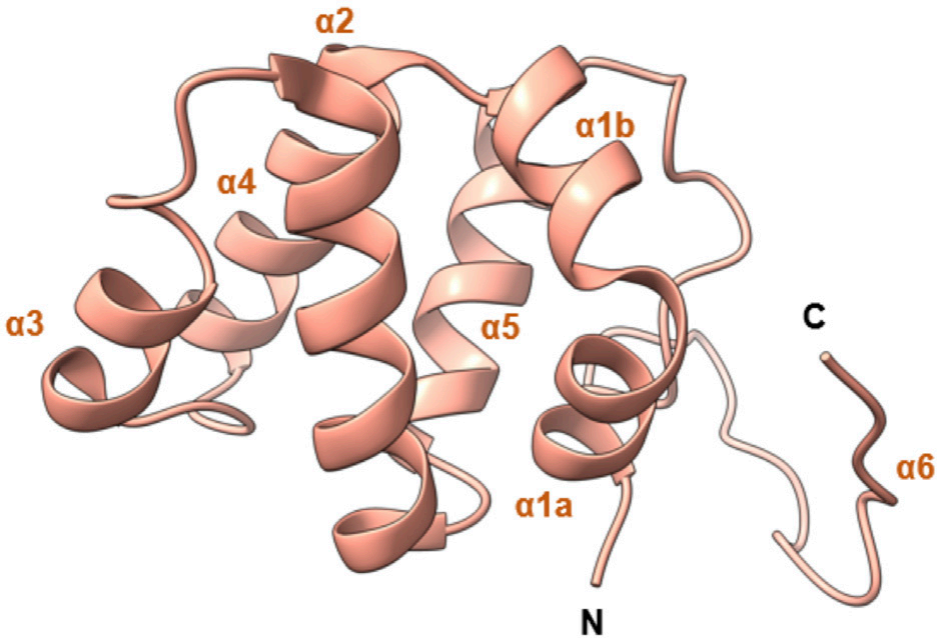
NMR solution structure of human RIPK2 CARD (PDB ID: 2N7Z).

Although CARD does not participate in the kinase functions of RIPK2, it is essential for RIPK2 activation by interacting with NODs (*vide infra*). The structure of RIPK2 CARD shares similar features with NOD1 CARD, including the arrangement of all but the last of the *a*-helices (Fridh and Rittinger, 2012; Lin et al., 2015; Maharana et al., 2015; Goncharuk et al., 2018; Maharana et al., 2021). The solution structure of human RIPK2 CARD contains six *a*-helices, that are closely packed (Figure 2) (Fridh and Rittinger, 2012; Lin et al., 2015). The helices in the middle of CARD (α2-α5) form a helix bundle by arranging in an antiparallel fashion. The helix α1 consists of two smaller helices (α1a, α1b), which are connected by a sharp kink (Fridh and Rittinger, 2012). The helix α6 is rather short (1 turn) and resides near the C terminus. The solution structure of rat RIPK2 CARD was also obtained by nuclear magnetic resonance (NMR) with minor differences compared to human RIPK2 CARD (Goncharuk et al., 2018). In both reported structures the CARD conformation is stabilized by backbone-backbone and sidechain-backbone H bonds. The core of CARD is highly hydrophobic, while the surface is hydrophilic with many charged residues, which can form salt bridges in CARD-CARD interactions (Fridh and Rittinger, 2012; Maharana et al., 2015; Maharana et al., 2021). Point mutations in CARD indicate that Arg444, Arg483, and Arg488 are crucial for NOD1 binding as they form ionic interactions with acidic residues of NOD1 CARD (Goncharuk et al., 2018).

### RIPK2 signaling in innate immunity

RIPK2 is expressed in antigen-presenting dendritic cells and macrophages, and plays a pivotal role in innate immunity as it participates in signaling through NODs (NOD1 and NOD2), and possibly TLRs (Kobayashi et al., 2002). While TLRs sense microbes at the cell surface or in endosomes, NODs recognize bacterial peptidoglycan (PGN) components in the cytoplasm (Caruso et al., 2014). Thus, RIPK2 takes part in both complementary defense mechanisms of the innate immune system. RIPK2 may also contribute to T cell proliferation and differentiation (Chin et al., 2002). However, the role of RIPK2 in adaptive immunity is still controversial (Hall et al., 2008). Moreover, the role of RIPK2 in TLR signaling remains less defined and requires further studies to resolve ongoing controversy (Kobayashi et al., 2002; Park et al., 2007; Hall et al., 2008). RIPK2 is broadly expressed in tissues including pancreas and placenta (Thome et al., 1998). However, its function beyond the immune compartment is much less understood. Therefore, in the following sections, we mainly summarize the canonical NODs/RIPK2/NF-κB and MAPK signaling pathways and their link to inflammatory diseases.

### NODs/RIPK2/NF-κB signaling pathway

**FIGURE 3:**
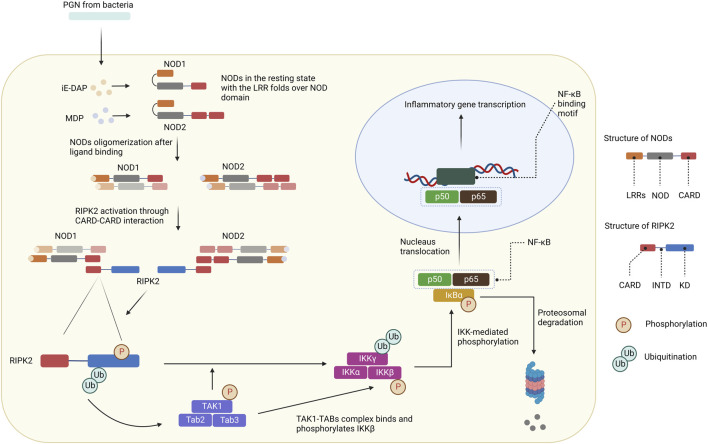
NODs/RIPK2 signaling pathways. After ligand binding, NODs oligomerize and form signaling complexes with RIPK2. RIPK2 is activated by auto-phosphorylation and further polyubiquitinated by E3 ligases. Ubiquitinated-RIPK2 turns on TAK1. pUb of both RIPK2 and IKKγ allows the TAK1-TABs complex to bind and phosphorylate IKKβ. IKK-mediated phosphorylation of the NF-κB inhibitor IκBα leads to its pUb and subsequent degradation through the UPS. Free NF-κB translocates to the nucleus and promotes the expression of downstream target genes for inflammatory cytokines and chemokines.

NOD1 and NOD2 are cytosolic NOD-like receptors. NOD1 is a scaffolding protein consisting of a CARD, a NOD domain, and multiple leucine-rich repeats (LRRs). While NOD1 is broadly expressed, NOD2 is expressed in monocytes and contains an additional CARD (Ogura et al., 2001). The NOD domain in NOD1 and NOD2 is composed of a nucleotide-binding domain (NBD), a winged helix (WH), and helix domains (HD1 and HD2). NOD1 is activated by γ-D-glutamyl-meso-diaminopimelic acid (iE-DAP), a common motif in many Gram^–^bacteria and some Gram^+^ bacteria (Girardin et al., 2003a; Chamaillard et al., 2003; Hasegawa et al., 2006), whereas NOD2 is activated by muramyl dipeptide (MDP) (Girardin et al., 2003b; Inohara et al., 2003; Hasegawa et al., 2006). In the resting state, NOD1 and NOD2 reside in an autoinhibited monomeric configuration in the cytosol with the LRRs folds over the NOD region (Hu et al., 2013). After peptidoglycans (PGN) is uptaken into cells, NOD1 and NOD2 are activated by direct ligand-receptor interactions (Lee et al., 2009; Grimes et al., 2012; Mo et al., 2012; Nakamura et al., 2014). The HD2 helix domain in the NOD module mediates conformational changes of the NBD, WH, and HD1 to allow ADP-ATP exchange (Tanabe et al., 2004). Binding to PGN components leads to NOD1 and NOD2 self-oligomerization, which forms signaling complexes with RIPK2 through homotypic CARD-CARD interactions (Figure 3) (Chen et al., 2009; Strober et al., 2014). As a result, RIPK2 is activated by auto-phosphorylation and subsequently polyubiquitinated by E3 ligases for non-degradative polyubiquitination (pUb) on Lys209 *via* Lys63-linked pUb (Hasegawa et al., 2008; Bertrand et al., 2009; Tao et al., 2009; Marinis et al., 2011; Yang et al., 2013; Zhang et al., 2014). The de-ubiquitinating enzyme A20 downregulates NODs/RIPK2 signaling by removing Lys63-pUb chains from RIPK2 (Wertz et al., 2004). Ubiquitinated-RIPK2 turns on the transforming growth factor-β (TGF-β)-activated kinase 1 (TAK1), which is necessary for the activation of the IκB kinase (IKK) complex and MAPK pathway (Hasegawa et al., 2008). The intermediate region and/or KD of RIPK2 is involved in the binding with IKKγ, whereas the CARD domain is dispensable (Inohara et al., 2000). IKKγ, a key component of the IKK signaling complex, is then ubiquitinated at Lys285 (Abbott et al., 2004). pUb of both RIPK2 and IKKγ allows the TAK1-TABs complex to bind and phosphorylate IKKβ to stimulate NF-κB (Abbott et al., 2007; Windheim et al., 2007; Yang et al., 2007; Hasegawa et al., 2008). IKK-mediated phosphorylation of the NF-κB inhibitor IκBα leads to its pUb and subsequent degradation through the ubiquitin-proteasome system (UPS). As a result, free NF-κB translocates to the nucleus and promotes the expression of downstream target genes for inflammatory cytokines and chemokines such as TNF-α, interleukin (IL)-6, IL-8, and membrane cofactor protein (MCP)-1.

### RIPK2/MAPK pathway

The MAPK signaling pathways regulate several cellular processes, including proliferation, differentiation, inflammation, and apoptosis (Zarubin and Han, 2005; Guo et al., 2020). There are four subgroups in MAPK family: ERKs, c-jun N-terminal or stress-activated protein kinases (JNK/SAPK), ERK/big MAP kinase 1 (BMK1), and the p38 group of protein kinases. Navas et al. (1999) have shown that RIPK2 is a Raf1-activated mitogen-activated Protein Kinase Kinase (MAPKK). RIPK2 kinase activity directly activates ERK1/2 and seems to mediate TNF-dependent ERK activation. After activation by GTP-Ras, Raf1 can phosphorylate RIPK2 and stimulate its activation of ERK2. RIPK2 can also activate p38 MAPK *via* TAK1 phosphorylation. The activation is achieved by the autophosphorylation of p38 MAPK after interaction with TAB1 (Jacquet et al., 2008). After being turned on, p38 MAPK can subsequently activate many transcription factors and lead to the production of proinflammatory cytokines, such as TNF-α, IL-1β, and IL-6.

## RIPK2 in inflammatory diseases

Inflammatory diseases refer to disorders and conditions characterized by inappropriate immune responses. Historically, immunotherapies relied on glucocorticoids and small drug molecules, which affect cell proliferation and metabolism (McInnes and Gravallese, 2021). Even though their efficacy has been proven, serious adverse reactions and the depletion of drug benefits over time call for safer and more effective therapies. Modern immunotherapies focus on precisely regulating immune responses (Kany et al., 2019; McInnes and Gravallese, 2021). Therefore, a deep understanding of the pathogenesis mechanisms is required. As one of the well-studied signaling pathways in innate immunology, the role of NODs/RIPK2 in multiple inflammatory diseases has been described in a number of reports. We highlight a few of the key findings as detailed below.

Negroni et al. (2009) have found that RIPK2 is overexpressed in inflamed tissues of CD patients. This observation could be linked to the increase of NOD2 since activation of the NOD2/RIPK2 pathway requires the formation of NOD2/RIPK2 immunocomplex. Stronati et al. (2010) also found an elevation of RIPK2 level in the colonic mucosa of UC patients, which involved both gene and protein expression. In a recent study, Watanabe et al. (2019) used plasmids expressing a RIPK2-specific siRNA to reduce disease severity in trinitrobenzene sulfonic acid (TNBS) induced colitis in mice. Knock-down of RIPK2 with siRNA also decreased activation of NF-κB, MAPK, and the accumulation of inflammatory cells. Interestingly, the RIPK2-specific siRNA also diminished TNBS induced colitis in both NOD2-deficient and NOD1/NO2-double deficient mice, indicating the effect of RIPK2 on colitis was independent of either NOD1 or NOD2 signaling. In agreement with this observation, the NOD1 levels only modestly increased, while NOD2 levels remained unchanged or even decreased in the endoscopic biopsy samples isolated from patients with IBD. In contrast, the RIPK2 expression was elevated compared to the control tissues.

RIPK2 also participates in the activation of CNS-infiltrating dendritic cells and is involved in the progression of multiple sclerosis (MS), a progressive neuroinflammatory disorder. To assess the role of RIPK2 in MS, Shaw et al. used the mouse experimental autoimmune encephalomyelitis (EAE) model by immunization with myelin oligodendrocyte glycoprotein (MOG) (Shaw et al., 2011). In RIPK2-deficient mice, the numbers of T cells in the central nervous system (CNS), as well as activated dendritic cells, were greatly reduced after MOG-induced EAE compared to the controls. Histological analysis of the spinal cord displayed a decrease in both inflammatory infiltrate and axon demyelination in *Ripk2* ^*−/−*^ mice. Hind-limb paralysis after EAE immunization was also reduced. Interestingly, the protection from disease progression was more consistent in *Ripk2* ^*−/−*^ mice compared to *Nod1* ^*−/−*^ or *Nod2* ^*−/−*^ mice. Furthermore, reconstitution with *Ripk2* ^*−/−*^ bone marrow-induced wild-type (WT) mice resistant to EAE.

Vieira et al. found that NOD2/RIPK2 signaling played essential roles in the development of experimental arthritis (Vieira et al., 2012). In the methylated bovine serum albumin (mBSA) induced arthritis mouse model, the contribution of RIPK2 to arthritis was highlighted by the reduction of neutrophil migration and mechanical hyper nociception in *Ripk2* ^*−/−*^ mice compared to the WT. In addition, the mBSA challenge did not cause cartilage erosion in *Ripk2* ^*−/−*^ mice. The authors also noticed a decrease in IL-17, TNF-α, and KC/CXCL1 chemokine production in RIPK2-deficient mice. Jurtnec et al. discovered the link between RIPK2 and osteoarthritis (OA) while sequencing the exomes of members of a family with an early-onset form of first metatarsophalangeal (MTP) joint OA (Jurynec et al., 2018). They found a rare missense variant in RIPK2 (rs200818100, c.310A>G, p.Asn104Asp) in affected individuals. This variant substitutes the common human Asn104 with Asp in the kinase domain of RIPK2. Since the N104D mutation results in a change of charge within the substrate binding site, RIPK2^104D^ has increased activity relative to RIPK2^104N^.

Behcet’s disease is a systemic inflammatory disease that lead to recurring ulcers in the mouth and other tissues (Ortiz-Fernández and Sawalha, 2021). A missense variant in RIPK2 (p.Ile259Thr) was recently identified as a susceptibility locus for Behcet’s disease in Turkish population (Takeuchi et al., 2017). Using induced gene expression in zebrafish embryos as a measure of the relative activities of RIPK2 alleles, Jurtnec et al. showed that RIPK2^259T^ significantly upregulated genes encoding NF-κB components, proinflammatory cytokines and genes downstream of proinflammatory cytokine signaling similar to RIPK2^104D^ (Jurynec et al., 2018).

Talreja et al. found the evidence for the contribution of RIPK2 to the pathology of sarcoidosis, a multisystem granulomatous disease that primarily occurs in the lungs (Talreja et al., 2016). In alveolar macrophages (AMs) and peripheral blood mononuclear cells (PBMCs) isolated from patients with sarcoidosis, RIPK2 expression was substantially higher compared to the controls. Pretreating Pam3CysSK4–TLR2 ligand (PAM) stimulated sarcoidosis AMs or PBMCs with a combination of IL-1 receptor-associated kinase (IRAK) 1/4 inhibitor and gefitinib (a non-selective RIPK2 inhibitor, *vide infra*) led to the reduction of IL-1β, IL-6 and INF-α productions. In agreement with this result, on genetic level, gefitinib and IRAK1/4 inhibitor inhibited IL-1β mRNA and RIPK2 mRNA levels. As a result, p38 activation was reduced as the MAPK pathway was blocked. Interestingly, neither inhibitor was effective as a single agent. This finding supports the hypothesis that both IRAK1/4 and RIPK2 participate in the pathogenesis of sarcoidosis.

## RIPK2 inhibitors

### Type I kinase inhibitors

Type I kinase inhibitors include ATP-competitors that occupy the ATP pocket of targeting kinase by mimicking the purine ring of ATP (Ferguson and Gray, 2018; Cohen et al., 2021; Ayala-Aguilera et al., 2022; Lee et al., 2022). Typically, type I kinase inhibitors contain a heterobicyclic aromatic ring that binds to the purine binding site within the active conformational state. Substituents of the aromatic core usually provide selectivity and high binding affinity for the inhibitors by occupying the hydrophobic region of the active site.

**FIGURE 4:**
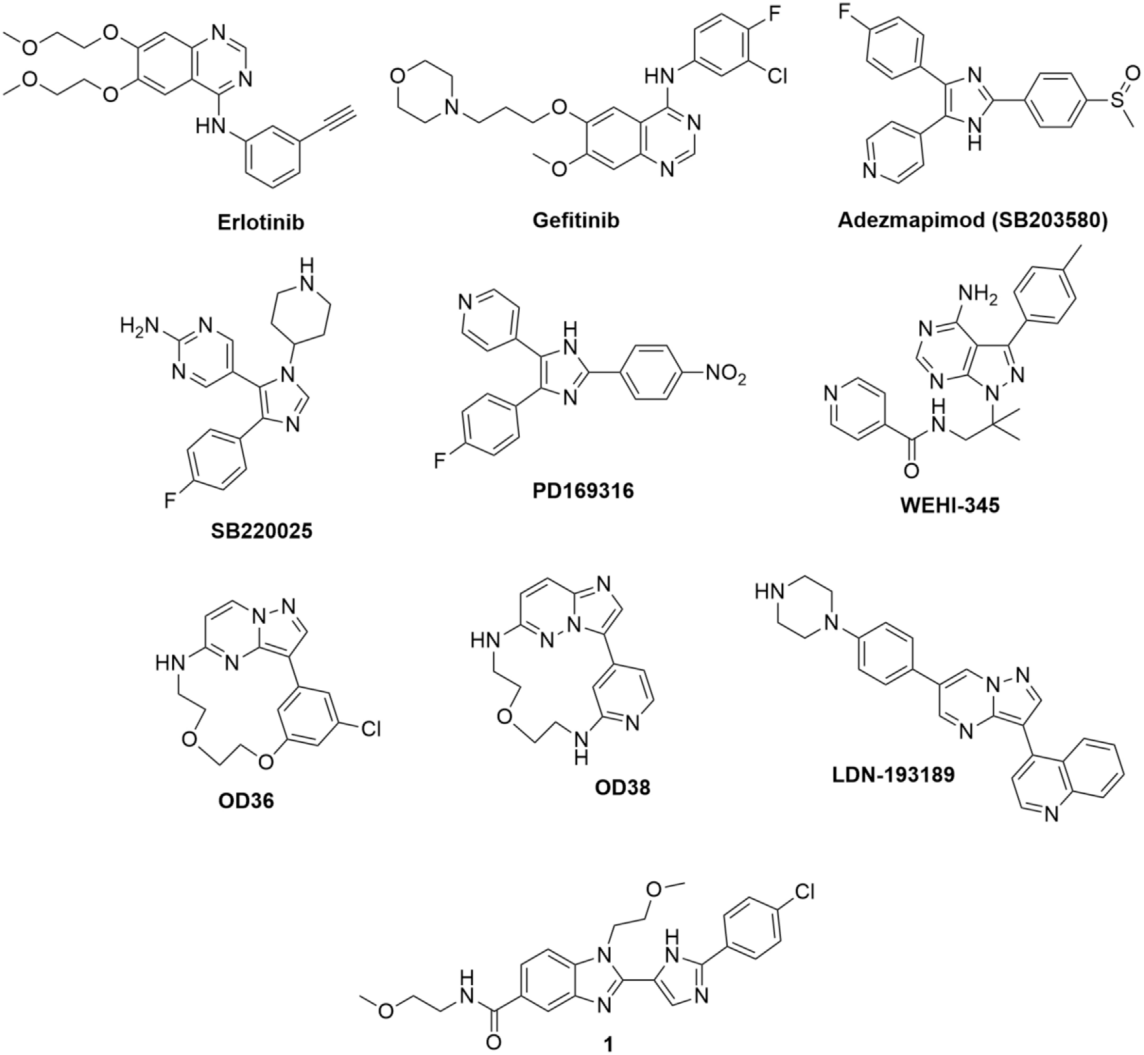
Structure of RIPK2 type I kinase inhibitors.

Tigno-Aranjuez et al. (2010) conducted a screening of compound library and identified erlotinib, gefitinib, and adezmapimod (SB203580) as RIPK2 inhibitors (Figure 4). Gefitinib and adezmapimod were also reported by others to inhibit RIPK2 activity (Argast et al., 2005; Brehmer et al., 2005; Hollenbach et al., 2005; Windheim et al., 2007). Erlotinib and gefitinib are type I tyrosine kinase inhibitors downstream of epidermal growth factor receptor (EGFR) singling, whilst adezemapimod is a p38 mitogen-activated protein kinase (MAPK) inhibitor. Erlotinib and gefitinib have been approved for the treatment of advanced and metastatic non-small-cell lung cancer, and their US Food and Drug Administration (FDA) and European Medicines Agency (EMA) particular public assessment reports (AR) can be obtained *via* the links provided here (FDA, 2016; EMA, 2023a; EMA, 2023b).

Erlotinib and gefitinib inhibited both tyrosine and serine-threonine phosphorylation of RIPK2, which subsequently inhibited RIPK2-induced IKKβ activation with similar efficacy. They acted as competitive inhibitors for ATP by binding to the ATP-binding pocket of their target kinase. In analogous to the EGFR T90M mutation that renders resistance to gefitinib (Kobayashi et al., 2005), the T95M point mutation of RIPK2 in the ATP binding pocket abolished the inhibitory activities of both erlotinib and gefitinib, indicating that these compounds directly inhibited RIPK2. The inhibition of RIPK2 tyrosine phosphorylation for both compounds occurred at nanomolar concentrations (IC_50_ for gefitinib = 51 nM). In addition, no inhibitory activities were noticed for TLR4 nor TNF signaling, demonstrating the specificity of erlotinib and gefitinib for the NOD2/RIPK2 pathway. Tigno-Aranjuez et al. further demonstrated the preclinical therapeutic efficacy of gefitinib for treating IBD (Tigno-Aranjuez et al., 2014). In SAMP1/YitFC mouse, a well-established mouse model of CD, the ileums and jejunums were evaluated after oral treatment with a 50 mg/kg/day dose of gefitinib. Villous height increasing and no acute inflammation at the border of the muscularis mucosa and lamina propria were found for the treated group compared to the controls.

Argast et al. reported that the autophosphorylation activity of RIPK2 *in vitro* was also blocked by adezmapimod (IC_50_ = 700 nM), its analogues SB220025 (IC_50_ = 390 nM), and PD169316 (IC_50_ = 70 nM) (Figure 4). Further investigation indicated that SB220025 and PD169316 behaved as ATP-competitive inhibitors, similar to adezmapimod (Argast et al., 2005). In colitis models, adezmapimod improved disease scores, reduced intestinal inflammation, and suppressed mRNA levels of pro-inflammatory cytokines (Hollenbach et al., 2005). TNBS-induced colitis mice treated with adezmapimod (5 μmol/kg, twice daily intraperitoneal injection) showed a lower body weight reduction. The macroscopic signs of inflammation in the colons and histological scores of treated mice were also reduced compared to the controls. In addition, the number of monocytes and the infiltration of macrophages, lymphocytes, and neutrophils decreased in the adezmapimod-treated animals. Adezmapimod also lessened the mRNA levels of inflammatory cytokines, such as TNF-α, interferon γ, IL-2, IL-12p35, IL-18, and IL-10 in the bowel tissue. From a high-throughput screening (HTS) campaign applying a fluorescence polarization (FP) based binding assay, Haile et al. also reported adezmapimod as a potent RIPK2 inhibitor (IC_50_ = 251 nM) (Haile et al., 2016).

In a screening campaign using a library of 120 kinase inhibitors, Nachbur et al. (2015) reported that WEHI-345, a pyrimidopyrazole analogue, bound to the ATP binding pocket of RIPK2 with IC_50_ = 130 nM. Docking of WEHI-345 into the murine RIPK2 homology model predicted that H bonds between the fused ring of WEHI-345 with Glu96 and Met98 in the hinge region of RIPK2 were crucial for the binding. The pyrimidopyrazole ring of WEHI-345 was sandwiched between Leu24 and Leu153, and formed hydrophobic effects with multiple amino acid side chains. The hydrophobicity of WEHI-345 seemed to contribute to the high affinity (K_D_) toward RIPK2 since more hydrophilic analogue showed significantly lower binding affinity. WEHI-345 showed high specificity for RIPK2 (K_D_: 46 nM) over other members of the RIPK family (K_D_: >10 μM for RIPK1, RIPK4, and RIPK5). Moreover, WEHI-345 only inhibited significantly (>90% at 1 μM) the activity of KIT, RET, PDGFRβ, and SRC among a panel of 92 kinases. Treatment with WEHI-345 significantly inhibited TNF-α and IL-6 secretion from MDP-simulated mouse bone marrow-derived macrophages (BMDMs) and Raw 267.4 cells. In *Listeria monocytogenes* infected CD11β^+^ monocytes, pretreatment with WEHI-345 also reduced cytokine and chemokine secretion. Consistent with its *in vitro* activity, WEHI-345 also normalized TNF and MCP-1 levels in MDP-stimulated mice. In induced chronic EAE C57BL/6 mice, treatment with WEHI-345 (20 mg/kg, twice daily intraperitoneal injection) significantly lowered the mean disease score compared to untreated or vehicle-treated mice. The reduction of inflammatory infiltrate was also detected in the forebrain of WEHI-345-treated animals. Furthermore, the authors observed overall improvement in disease progression as treated mice showed improved body weight and reduction of cytokine and chemokine levels.

Tigno-Aranjuez et al. (2014) identified two potent RIPK2 inhibitors, OD36 and OD38, from a screening of macrocyclic small molecules. These compounds mimicked the interactions between the ATP adenine ring and the hinge region of RIPK2 KD. Besides, these novel inhibitors also utilized the interactions between the cyclic linker and the hydrophobic back pocket and the ribose binding region to improve the affinity and selectivity toward RIPK2. OD36 and OD38 displayed very potent activity against RIPK2 with IC_50_ of 5.3 nM and 14.1 nM, respectively. They also showed reasonable initial specificity and only inhibited a few other kinases at nanomolar concentrations in a 366-kinase panel. In BMDMs isolated from SAMP1/YitFc CD mouse model, OD36 and OD38 downregulated various MDP-induced inflammatory cytokines and chemokines as well as expression of NOD2 and RIPK2. From qRT-PCR analysis results, the authors proposed a genetic RIPK2 activation panel of 9 genes (*GPR84, ICAM1, IRG1, MARCKSL1, CD40, RASGRP1, SLC2a6, CLEC4e, CXCL10*), which could be used to determine if a patient is responsive to RIPK2 inhibitor treatment.

**FIGURE 5:**
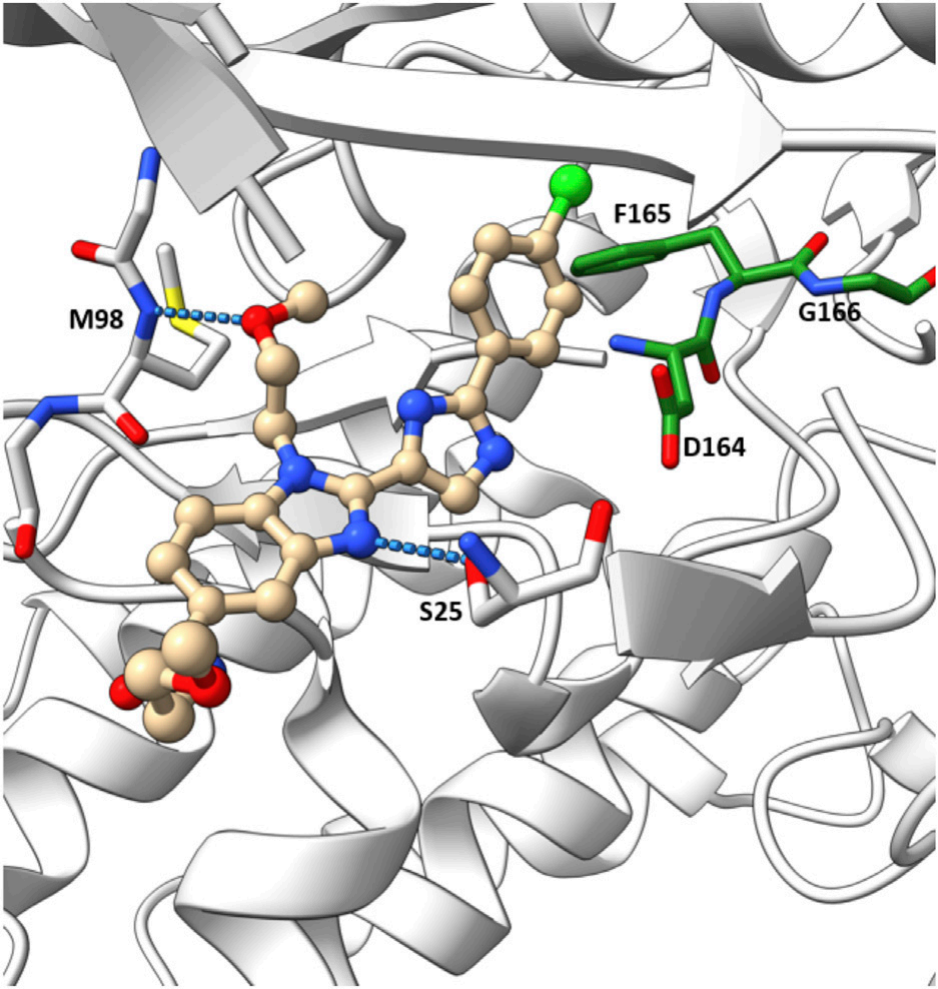
Co-crystal structure of **1** and RIPK2 (PDB ID: 5AR5). Ligand-receptor H-bonds are indicated by dashed lines. The DFG motif is shown in green.

In their crystallographic study Charnley et al. from GlaxoSmithKline (GSK) described a novel RIPK2 inhibitor chemotype–the benzimidazole analogue 1 (IC_50_ = 500 nM) through GSK encoded library technology (ELT) collection (Figure 4) (Charnley et al., 2015). 1 displayed a unique binding mode to the ATP binding pocket of RIPK2 with an H bond between the benzimidazole N1-methylether O atom and backbone NH of Met98, and another H bond between the benzimidazole N3 and sidechain OH of Ser25 (Figure 5). The imidazole-phenyl group with the chlorine substituents occupied the hydrophobic back pocket. In a 352-kinase panel, 1 inhibited only 6 kinases by >70% at 10 μM. The high selectivity of 1 was thought to result from the unique binding mode. However, 1 exhibited rapid dissociation from RIPK2 in binding kinetics studies (K_off_ = 0.12 s^-1^, T_1/2_ = 0.1 min) indicating transient target engagement.

**FIGURE 6:**
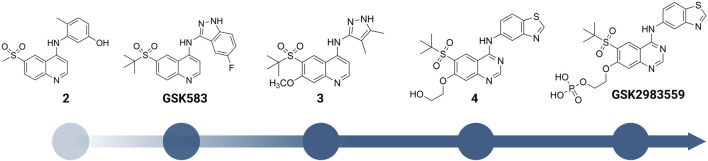
Structure modification of **2** and **GSK583**. **GSK2983559** was the first RIPK2 inhibitor which entered clinical studies.

**FIGURE 7:**
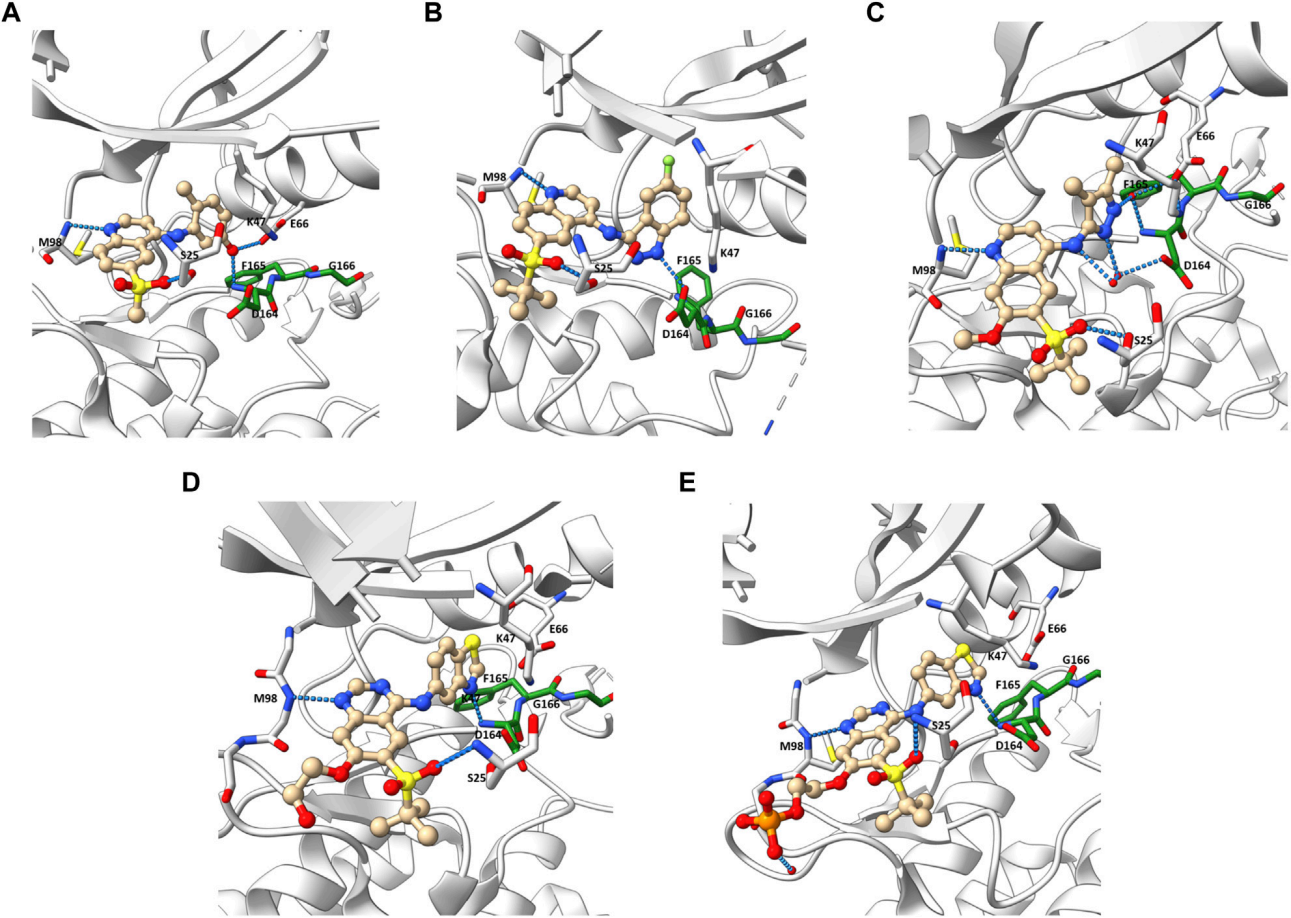
Co-crystal structure of RIPK2 in complex with **2** (PDB ID: 5J79) **(A)**, **GSK583** (PDB ID: 5J7B) **(B)**, **3** (PDB ID: 6HMX) **(C)**, **4** (PDB ID: 6RNA) **(D)**, and **GSK2983559** (PDB ID: 6RN8) **(E)**.

A more dedicated RIPK2 inhibitor discovery campaign was launched by GSK soon after their initial study (Haile et al., 2016). By using a fluorescence polarization (FP) based binding assay for screening the GSK kinase inhibitor set and utilizing ELT in an affinity screen, Haile et al. identified the quinoline derivative 2 (Figure 6) as a novel RIPK2 inhibitor (IC_50_ = 3 nM). The co-crystal structure with RIPK2 showed that 2 occupied the ATP-binding pocket in a traditional type I kinase inhibitor fashion, with the quinoline core sitting in the adenine binding pocket (Figure 7A). In addition, the quinoline nitrogen provided a hinge interaction with the backbone N-H of Met98. An H bond was found between the sulfone oxygen and Ser25. The phenol group also formed H bonds with Glu66 and Asp164.

2 was moved forward to the hit-to-lead optimization phase and applied to structure-based drug design (SBDD) (Figure 6). From the co-crystal structure, the authors realized that the large and flexible hydrophobic back pocket of RIPK2 provided an opportunity to optimize the structure of 2 to achieve better kinase selectivity. The ELT affinity screening of a 4-substituted quinoline containing a library of >400 anilines resulted in the identification of 5-aminobenzothiazole and 5-aminobenzotriazole as the best substituents to be incorporated into the structure of 2 to form strong RIPK2 binders. However, to improve the kinase selectivity, the 3-aminoindazole group was chosen despite lower potency. Several substituents at the C6-position of the quinoline ring were also screened. Nonetheless, the initial sulfone group showed the best balance of potency, physicochemical properties, permeability, and lipophilic efficiency (LipE). Finally, the steric effect of the sulfone group at C6-position was examined by modifying the methyl group to isopropyl or *tert*-butyl groups. Moderate improvements in binding affinity were observed with GSK583 having *tert*-butyl substituent (IC_50_ = 5 nM); it was 10-fold more potent than its methyl-substituted analogue (IC_50_ = 50 nM).

GSK583 exhibited excellent selectivity in a panel of 300 kinases, in which only two additional kinases were found to be inhibited by approximately 30%. The co-crystal structure of GSK583 with RIPK2 displayed a similar binding mode to the initial screening hit 2 (Figure 7B). The indazole moiety sat in the back pocket, created hydrophobic effects with the roof of the pocket, and formed an H bond with Asp164.

The potency of GSK583 was evaluated across multiple assays. In normal primary human monocytes, GSK583 inhibited MDP-stimulated TNF-α production with IC_50_ = 8.0 nM. Likewise, in a similar assay in human whole blood, the IC_50_ was reasonable at 237 nM. GSK583 also exhibited significant inhibition in TNF-α and IL-6 productions in human CD and UC biopsy assays (IC_50_ ∼ 200 nM). Despite the potency of GSK583 *in vitro*, poor pharmacokinetic (PK) parameters were observed *in vivo* for both rats and mice. GSK583 showed low clearance, moderate volumes of distribution, and moderate oral bioavailability (Table 1). In addition, off-target activity against the hERG ion channel (IC_50_ = 7445 nM) was later noticed for GSK583 (Haile et al., 2018). Hence, this molecule was excluded from further development as a drug candidate.

**TABLE 1:** PK parameters of selected RIPK2 inhibitors.

Compound	Species	Oral/iv dose (mg/kg)	Oral C_max_ (ng/mL)	Oral AUC (ng.h/mL)	Cl (mL/min/kg)	t_1/2_ (h)	%F
GSK583	rat	2.0/1.0	260	980	15	3.1	39
GSK583	mouse	2.0/1.0	340	860	13	2.1	34
3	rat	1.1/2.0	740	2400	12	3.1	85
3	dog	1.1/2.0	330	1900	20	2.5	96
4	rat	2.0/0.5	120	460	27	4.4	39
4	dog	2.0/1.1	470	1800	9.9	5.1	57
4	minipig	1.0/0.4	31	85	18	1.9	26
GSK2983559^a^	rat	2.0/1.1	170	560	30	2.4	77
GSK2983559^a^	dog	2.0/0.9	840	4400	11	2.9	100
GSK2983559^a^	minipig	2.0/1.0	340	1100	11	4.2	58
8	mouse	10.0/2.0	1638	3351	26.1	1.0	47
8	rat	20.0/5.0	5992	38469	12.6	3.2	130
8	dog	2.0/0.5	987	6487	7.8	5.0	135
BI 706039^b^	mouse	0.3/1.0	517	2374	ND	ND	ND

Notes: iv, intravenous; C_max_, maximum plasma concentration; AUC, area under the curve; Cl, clearance; t_1/2_, half-life; F, bioavailability.

^a^ PK, parameters of 4 following oral administration of prodrug GSK2983559.

^b^ : C_max_ in nM, and AUC in nM.h. ND: not determined.

In a subsequent study, Haile et al. addressed the existing problems of GSK583 by eliminating aromatic groups from GSK583 structure, which resulted in compound 3 as a more suitable lead compound (RIPK2 FP IC_50_ = 1.3 nM) (Figure 6) (Haile et al., 2018). A ring contraction strategy was applied to maintain the excellent potency while reducing its lipophilicity. Replacing the benzo-fused indazole ring in GSK583 with two methyl groups at the 3- and 4-positions of the pyrazole ring kept the essential hydrophobic interactions of GSK583 with the back pocket of RIPK2, as demonstrated by the co-crystal structure of RIPK2 in complex with 3 (Figure 7C). An electron-donating group (MeO) was introduced to C7-position of the 4-aminoquinoline template in 3 to strengthen the hinge binding interaction between N1 and Met98. Gratifyingly, these modifications reduced the activity of 3 against the hERG channel (IC_50_ = 14500 nM). Compound 3 exhibited a desired greater than 100-fold window between the human whole blood RIPK2 inhibition potency and inhibition of the hERG channel.

The oral availability of 3 (85% in rat) was enhanced compared to GSK583 (39% in rat) (Table 1). 3 had overall a better PK profile in comparison with GSK583 as seen by substantially higher maximum blood concentration (C_max_) and area under the curve (AUC) (Table 1). 3 was also effective to reduce cytokine levels *in vivo* in an acute MDP-simulated rat peritonitis model with IC_50_ = 19 nM.

Scaffold hopping (i.e., quinoline to quinazoline; indazole to benzothiazole) resulted in the 4-aminoquinazoline series (4, Figure 6; Figure 7D) that had a divergent structure-activity relationship (SAR) in inhibitory activities for RIPK2 and hERG ion channel (Haile et al., 2019). The quinazoline series exhibited a better safety profile compared to 3 as almost no hERG inhibitory activity was detected (IC_50_ > 30 μM). 4 was reported as the lead molecule in these studies (IC_50_ = 5 nM) with reasonable PK parameters in rat, dog, and minipig (Table 1). However, high dose safety assessment studies in rats showed a lack of dose-exposure linearity, which was likely caused by poor solubility in fasted state simulated intestinal fluid. Therefore, 4 likely precipitated in the gastrointestinal (GI) tract as the dose increased. Since various formulation strategies failed to solve this problem, a prodrug approach was utilized by introducing a phosphate group to the free hydroxyl of 4, leading to the phosphoric acid GSK2983559 and its salt forms with improved the compound’s solubility (Figure 6). After cleavage of the phosphate group in GI tract, the parent compound 4 was released and quickly absorbed. PK studies showed efficient delivery of compound 4 after oral administration across species (Table 1). Meanwhile, the efficacy of 4 was validated using biochemical, cellular, and tissue-based functional assays. All of them showed a high affinity of 4 toward RIPK2. In HEK293 cells overexpressed NOD2, 4 potently inhibited MDP-simulated production of IL-8 (IC_50_ = 4 nM). In monocytes, TNF-α production was potently inhibited by 4 (IC_50_ = 13 nM). Prodrug GSK2983559 exhibited similar potency in biochemical assays as the phosphate group was directed toward the solvent front upon binding to RIPK2 (Figure 7E). In mucosal biopsy samples obtained from CD or UC patients, 4 reduced productions of both IL-1β and IL-6 in a dose-dependent manner.

Prodrug GSK2983559 displayed excellent *in vivo* efficacy in a murine TNBS-induced colitis model. Based on overall colon scores, GSK2983559 showed a comparable effect at 7.5 and 145 mg/kg b.i.d doses compared to the standard control prednisolone. The improved solubility and dose linearity in oral administration allowed GSK2983559 to advance to clinical trials for IBD (ClinicalTrials.gov Identifier: NCT03358407) (Haile et al., 2019).

**FIGURE 8:**
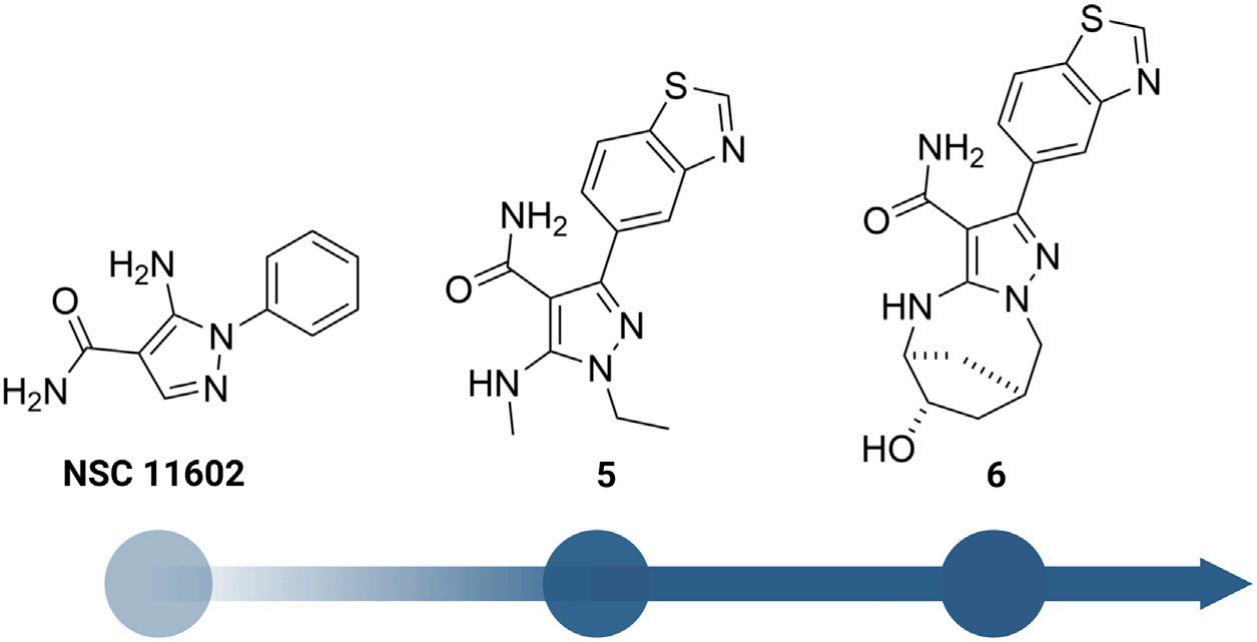
Structure optimization of GSK’s pyrazole derivatives.

**FIGURE 9:**
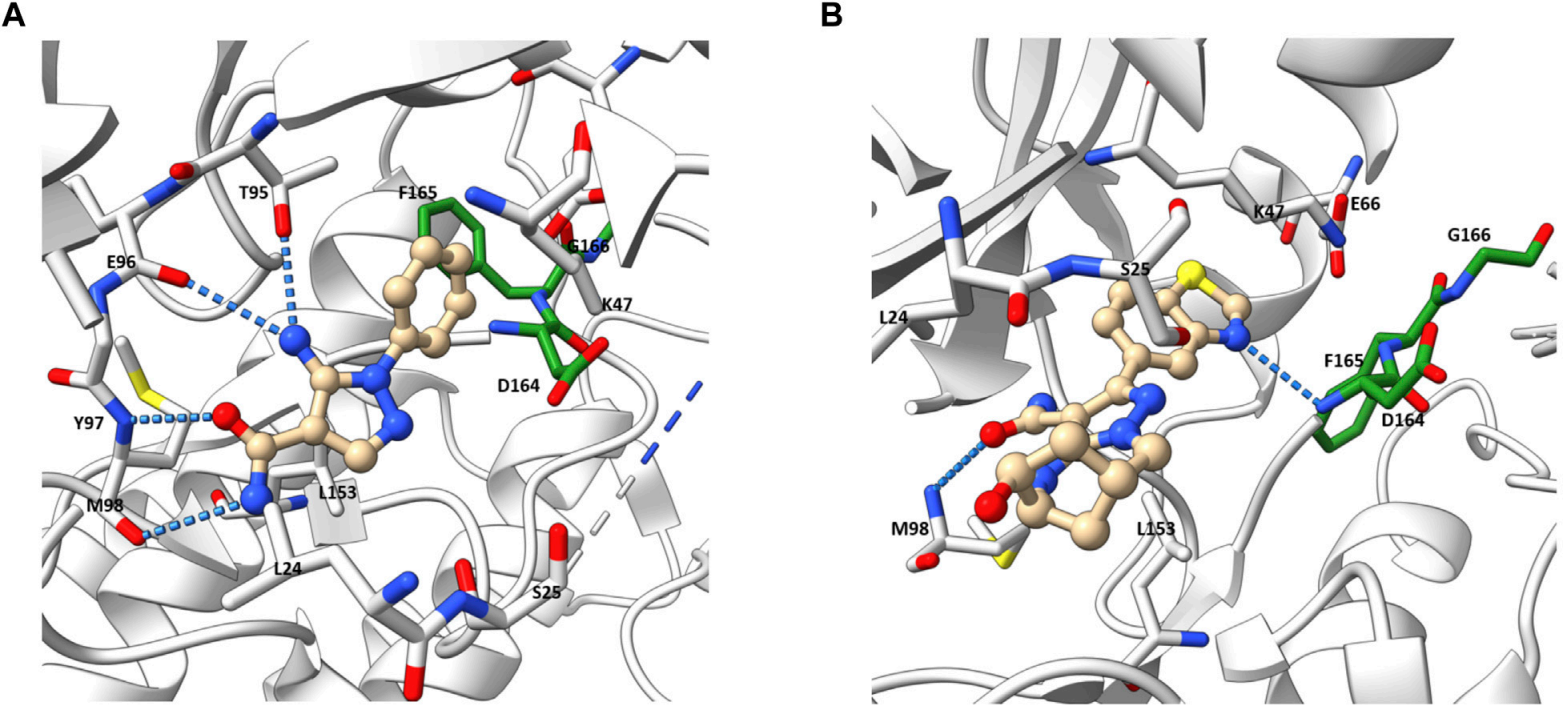
Co-crystal structure of RIPK2 in complex with **NSC 11602** (PDB ID: 6SZE) **(A)** and **6** (PDB ID: 6UL8) **(B)**.

Besides the quinazoline-based series, scientists at GSK also explored other chemical scaffolds to develop novel RIPK2 inhibitors. From a fragment-based screening and design program, Haffner et al. (2019) identified 30 hits by a thermal shift assay. After filtering potential structures by crystallography, the authors narrowed the program to a single chemical series derived from pyrazolocarboxamide NSC 11602 (IC_50_ = 80 μM) (Figure 8). Interestingly, the introduction of substituents at C3-position of the pyrazole ring (as in 5) flipped the molecules in the ATP binding pocket of RIPK2 relative to the initial fragment NSC 11602 (Figure 9). The C3 aryl group of the novel derivatives occupied the back pocket and reversed the hinge and gatekeeper interactions (Figure 9B). Non-etheless, the inhibitory activity of 5 to RIPK2 was greatly improved (IC_50_ = 60 nM). To achieve better selectivity, the hit compound 5 was further optimized to produce 6 as the lead compound in this study. The bridged bicyclic ring in 6 was shown by crystallographic study to provide additional hydrophobic interactions with Leu24 and Leu153, while the hydroxyl group is pointed to solvent and not involved in interactions with the kinase (Figure 9B). Although RIPK2 binding affinity for 6 was only 2-fold improved (IC_50_ = 30 nM) compared to 5, the selectivity of 6 against three key essential kinases: activin receptor-like kinase 5–ALK5, vascular endothelial growth factor receptor 2–VEGFR2, and lymphocyte-specific protein tyrosine kinase–LCK was improved with all IC_50_ > 5.0 μM.

**FIGURE 10:**
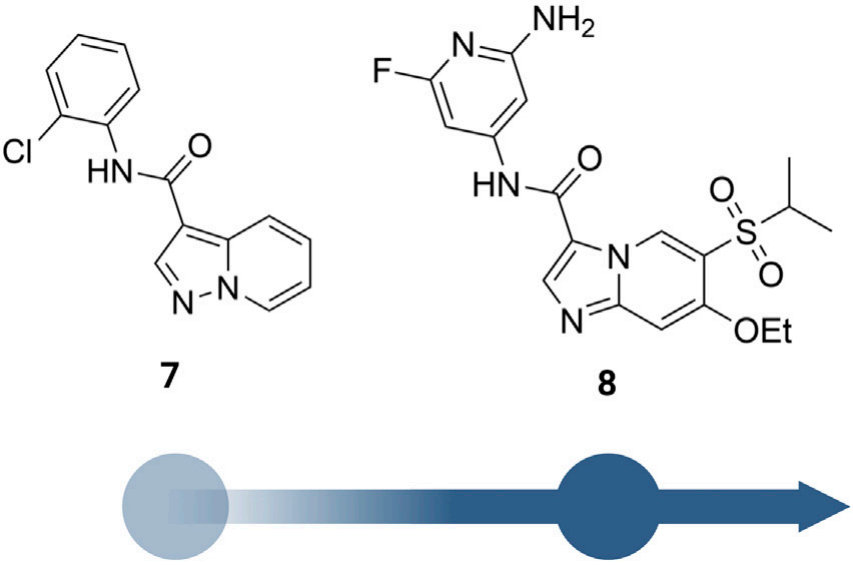
Structure optimization of Norvatis’s RIPK2 inhibitors.

He et al. from Novartis reported their lead identification and optimization program for the discovery of RIPK2 inhibitors (He et al., 2017). A virtual library screen was performed with ∼11 million compounds. After hits selection and flexible docking to RIPK2 protein structure, approximately 10,000 virtual hits were clustered and analyzed. A RIPK2 homogenous time-resolved fluorescence (HTRF) ADP transcreener assay, which measures kinase activity by following the formation of ADP using an anti-ADP antibody labeled with europium (Eu) and a d2-labeled ADP (tracer) (Hong et al., 2009), was applied to confirm the activity of virtual hits. Results suggested compound 7 (Figure 10) as the most suitable for further characterization (IC_50_ = 1,500 nM).

**FIGURE 11:**
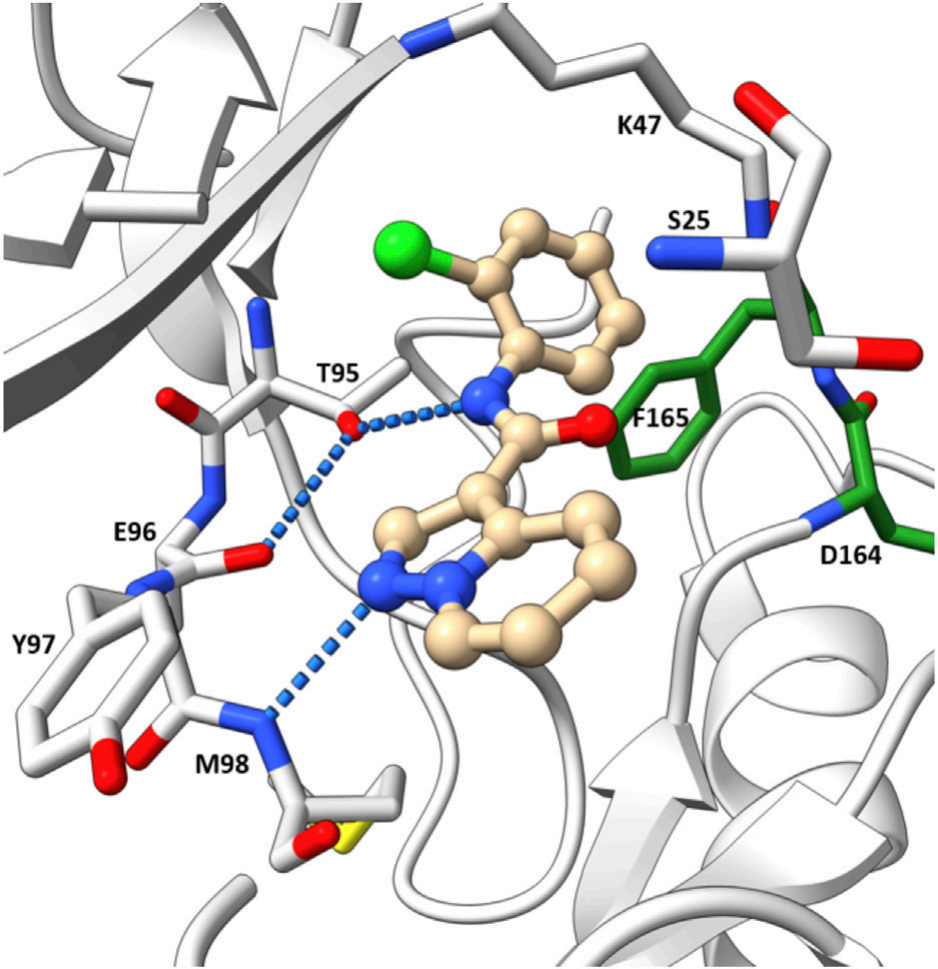
Co-crystal structure of **7** and RIPK2(PDB ID: 5W5J).

The co-crystal structure revealed that 7 binds to the ATP binding site in a typical type I kinase inhibitor fashion with the DFG motif adopting a DFG-in conformation (Figure 11). 7 formed a hinge interaction between N1 and Met98. The gatekeeper Thr95 provided an additional H bond with the amide N-H. The phenyl ring fitted into the back pocket, with the chlorine atom occupying a small hydrophobic pocket.

SAR studies led to the discovery of imidazo [1,2-a]pyridine as a better core than pyrazolo [1,5-a]pyridine. Similar to the GSK compounds, an alkyl sulfone group was placed at C5-position, and an ethoxy group was incorporated at C6-position of the aromatic core, leading to 8 that exhibited a much higher affinity and selectivity for RIPK2, with IC_50_ = 3 nM in the RIPK2 ADP transcreener assay. In the structure of 8, the aryl carboxamide group at C3-position provided a good balance between potency and stability, and the isopropyl group together with the ethoxy served to optimize the metabolic stability. 8 displayed high stability, low clearance, and good oral exposure in mice, rat, and dog (Table 1). Kinase selectivity of 8 was confirmed in a 250-kinase panel. At 0.5 μM (>100-fold of RIPK2 IC_50_), 98% of kinases were inhibited by less than 50%. In MDP and LPS-stimulated mouse BMDMs, 8 suppressed the secretion of IL-6 in a dose-dependent manner with IC_50_ = 12 nM. In both rat and human whole blood stimulated by MDP, 8 also effectively reduced TNF-α production. In a rat *ex vivo* PK/PD study, 8 inhibited MDP-induced cytokine secretion in blood at all time points. Furthermore, *in vivo* studies in rats showed that 8 could reverse MDP-induced proinflammatory cytokines in the colons. In blood, a significant reduction in IL-6 and TNF-α levels was observed.

**FIGURE 12:**
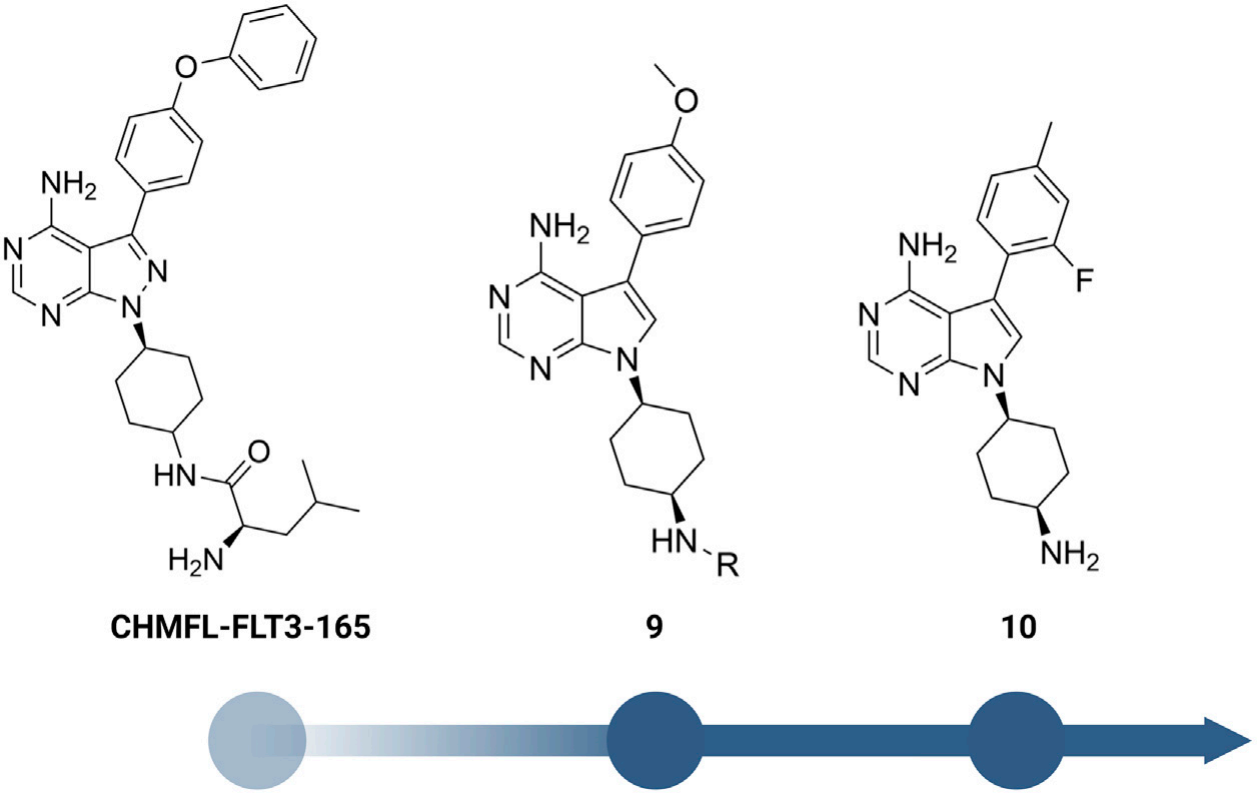
Structure optimization of **CHMFL-FLT3-165**.

Yuan et al. designed a new series of RIPK2 pyrrolopyrimidine inhibitors based on structural modification of the FLT3 inhibitor CHMFL-FLT3-165 (Figure 12) (Wu et al., 2016; Yuan et al., 2022). CHMFL-FLT3-165 strongly inhibited RIPK2 and RIPK5 at 1 μM concentration. Docking of CHMFL-FLT3-165 into the RIPK2 model revealed that the amino group of pyrrolopyrimidine, an adenosine mimetic, formed an H bond with Thr95 and Asp164. In addition, the oxygen of diphenyl ether was crucial to establish another H bond with Met98. Based on docking results, Yuan et al. designed a new series of RIPK2 inhibitors 9, which retained critical functional groups for kinase binding (Figure 12). Even though the initial design led to a series of nM active RIPK2 inhibitors (IC_50_ ranged from 3.0 to 21.1 nM) with high selectivity over RIPK1 and FLT3 (less than 60% of inhibition at 1.0 μM of 9), these compounds suffered from fast clearance (from 220.8 to 885.4 mL/min/g.protein). Screening substituents at 3-position provided compound 10 with exceptional potency (IC_50_ = 0.6 nM), improved PK, and low toxicity. 10 showed good clearance (<12 mL/min/g.protein) and lower toxicity in H9C2 cells. Consistent with the high affinity toward RIPK2, 10 potently inhibited TNF-α production in MDP-stimulated BMDMs. Further *in vivo* experiments on a colitis model induced by dextran sodium sulfate showed that 10 was more effective in minimizing weight loss and colon tissue damage than the RIPK2 inhibitor WEHI-345 and the Janus tyrosine kinase (JAK) inhibitor filgotinib (approved in the United Kingdom for the treatment of UC).

A series of novel thienopyridine derivatives were disclosed as RIPK2 inhibitors in a patent application (WO 2020/132384) by Celgene (acquired by Bristol-Myers Squibb). Sabnis provided a concise summary of the disclosed chemical matters (with a general formula I) and highlighted a number of derivatives with IC_50_ < 10 nM (92).


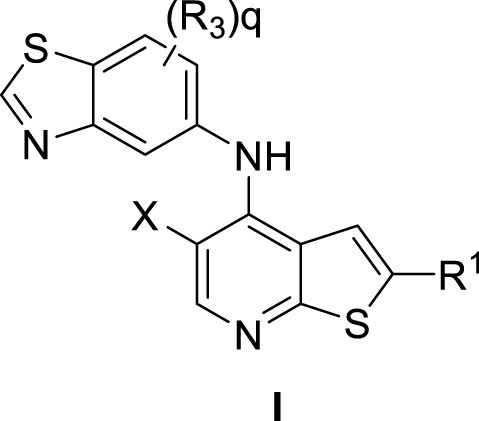


Boehringer-Ingelheim also reported a RIPK2 inhibitor—BI 706039 (structure not disclosed), which was discovered by optimization of HTS hits (Ermann et al., 2021). BI 706039 was highly selective and only inhibited 18 kinases in a 285-kinase panel at 3.0 μM. In human monocyte, BI 706039 fully blocked the production of MDP-induced TNF-α. Similarly, in a mouse whole blood assay, BI 706039 inhibited the murine INFγ and MDP-stimulated TNF-α production with IC_50_ of 4.5 nM. BI 706039 showed promising PK properties and good oral bioavailability (Table 1). The efficacy of BI 706039 was further probed in the TRUC mouse model of IBD. Oral administration of BI 706039 significantly improved the colonic histopathology of treated mice. In addition, the lipocalin ratio was decreased in a dose-responsive manner. Treating mice model with BI 706039 also downregulated the expression of a range of inflammatory disease-associated genes, such as *Tnf, Tnfaip3, Nod2, and Ccl20*. A clinical study on the efficacy of BI 706321 in combination with Ustekinumab for the treatment of CD is ongoing (ClinicalTrials.gov Identifier: NCT04978493).

### Type II kinase inhibitors

In contrast to type I kinase inhibitors, type II kinase inhibitors target the inactive conformation of kinases by interacting with the catalytic site of unphosphorylated proteins (Bhullar et al., 2018). In the inactive conformation, the DFG motif of the kinase is directed away from the ATP-binding site. The exclusivity of inactive kinase conformations renders type II kinase inhibitors more selective than type I. However, type II kinase inhibitors for RIPK2 are still scarce, with a few identified from HTS of known kinase inhibitors and no drug discovery campaigns to optimize their structures have been reported.

**FIGURE 13:**
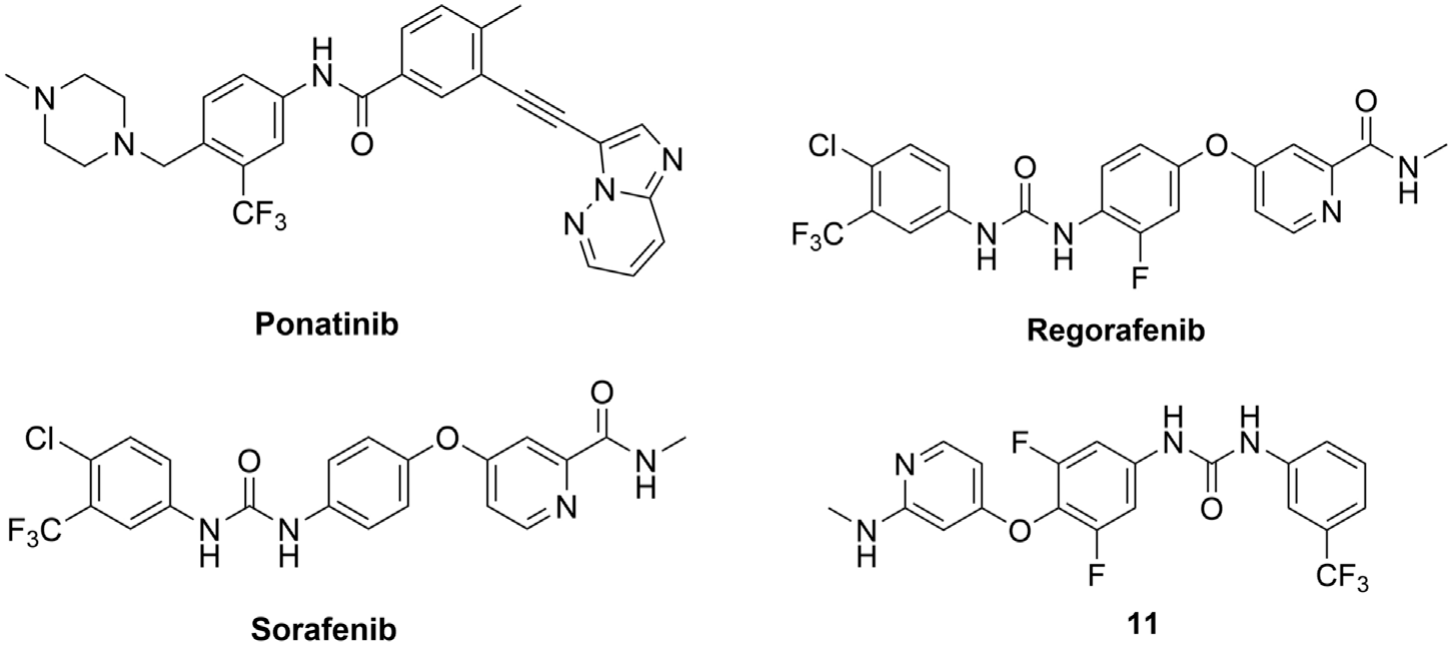
Structures of type II RIPK2 kinase inhibitors.

In 2015, Canning, Ruan et al. used a fluorescence-based thermal shift assay to identify several type II RIPK2 inhibitors–ponatinib (IC_50_ = 6.7 nM), regorafenib (IC_50_ = 41 nM), and sorafenib (IC_50_ = 75 nM) (Figure 13) (Canning et al., 2015). In THP-1 cells, pre-treatment with ponatinib (100 nM) completely inhibited L18-MDP-induced RIPK2 ubiquitination. Consistent with this result, in MDP-stimulated RAW264.7 macrophage cells, low nanomolar (1–10 nM) concentrations of ponatinib potently decreased mRNA levels of CCL4, CXCL2, and RANTES.

**FIGURE 14:**
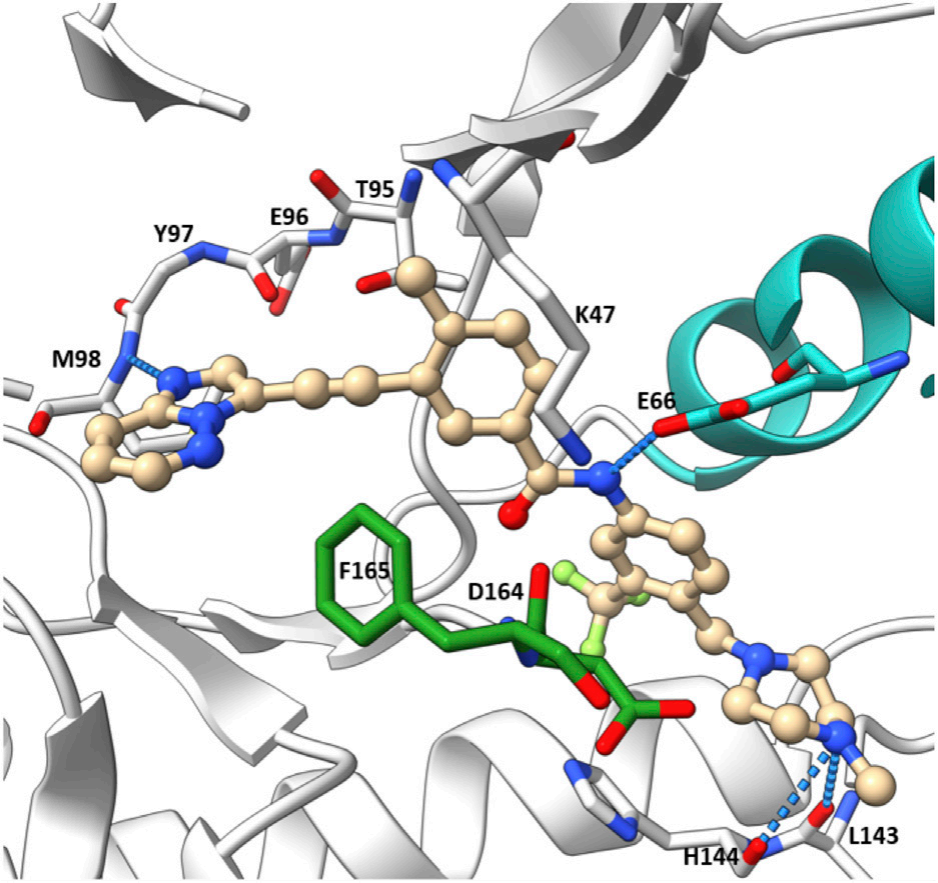
Co-crystal structure of **ponatinib** and RIPK2 (PDB ID: 4C8B).

The co-crystal structure of RIPK2 with ponatinib showed that it occupied the ATP pocket of inactive RIPK2 with the “DFG-Asp out, αC-Glu in” configuration, characteristic of kinases in an inactive conformation (Figure 14) (Schwarz et al., 2019). The imidazo [1,2-b]pyridazine head group formed an H bond to the hinge residue Met98 as well as hydrophobic effects with Tyr97 and Leu24. The central linker provided two additional H bonds with the side chain of Glu66 and the main-chain nitrogen of Asp164. The trifluoromethyl group sat in the hydrophobic pocket of the DFG motif, while the protonated methyl piperazine formed an ionic-dipole interaction with the main-chain oxygen of Leu143 and His144. In RIPK2, the hydrophobic pocket occupied by the CF_3_ group is larger than most other kinases, which suggested that replacing CF_3_ in ponatinib with bigger groups could generate RIPK2-selective inhibitors. Regorafenib and sorafenib were suggested to bind to RIPK2 similarly despite lower potency *in vitro* (Canning et al., 2015). Additionally, compound 11 (Figure 13), a sorafenib analogue, was described as a screening hit (RIPK2 FP IC_50_ = 794 nM) (Haile et al., 2016). But it is unclear if 11 binds to RIPK2 in the same fashion as ponatinib.

Even though these type II kinase inhibitors are approved anti-cancer medications (their corresponding AR can be accessed by following the links for ponatinib (EMA, 2013a), regorafenib (EMA, 2013b), and sorafenib (EMA, 2007)), the broad kinase selectivity of these drugs prohibited their application as RIPK2 inhibitors in clinics. Nonetheless, they provided a promising starting point for developing selective type II RIPK2 inhibitors.

### RIPK2 PROTACS

Proteolysis-Targeting Chimera (PROTAC) is a rapidly emerging technology that hijacks the ubiquitin-proteasome system (UPS) to degrade the proteins of interest (POI). PROTACs are hetero-bifunctional molecules that simultaneously bind to a POI and an E3 ligase, which leads to ubiquitination of the POI and its subsequent degradation by UPS. After the POI is degraded, PROTAC is recycled and continues to bind to a new copy of the POI. This catalytic nature of PROTACs distinguishes them from traditional inhibitors (Békés et al., 2022; Hu and Crews, 2022).

**FIGURE 15:**
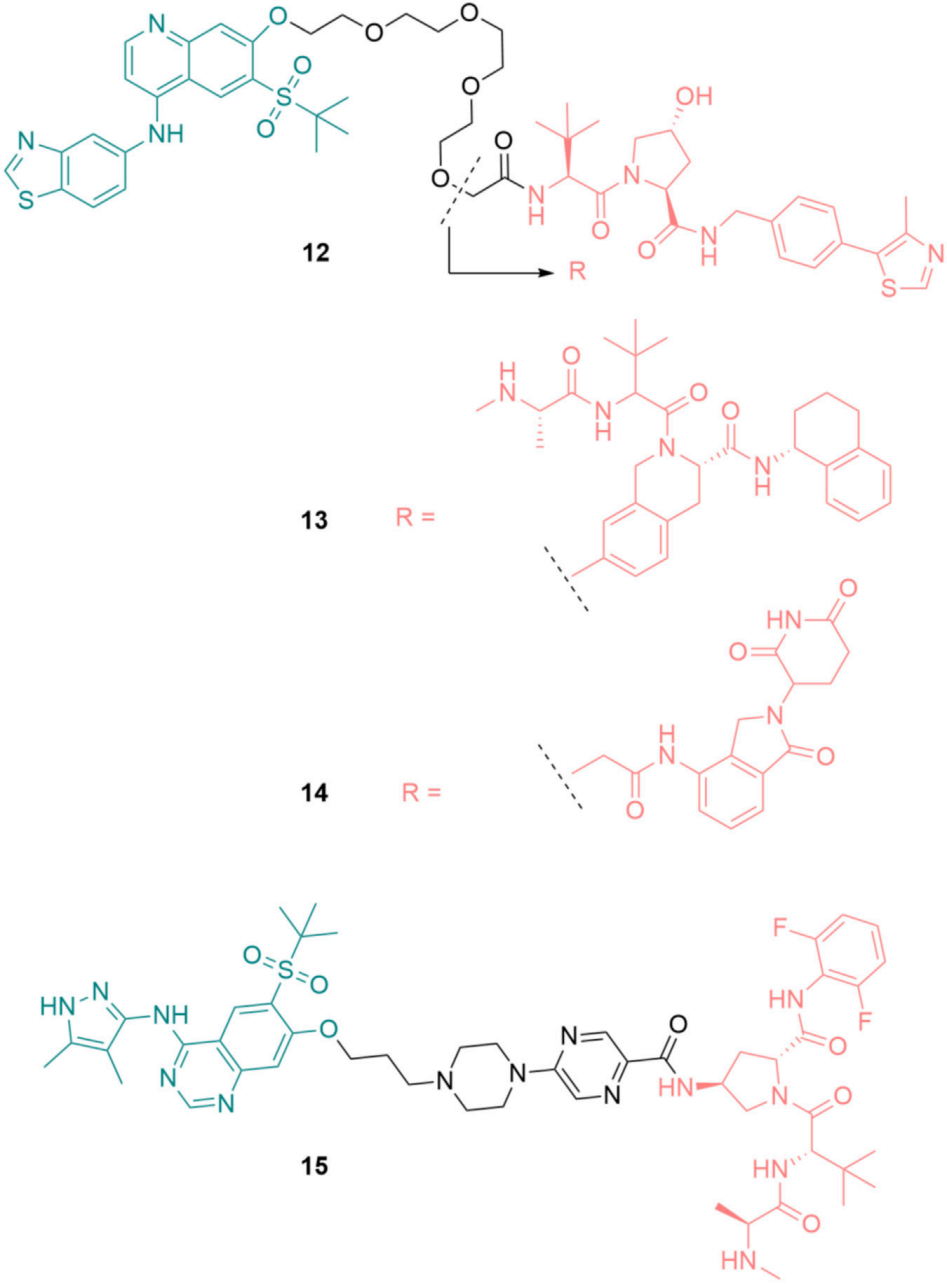
Structures of RIPK2 PROTACs. RIPK2 binders are highlighted in green. E3 ligase binders are highlighted in red.

Based on the RIPK2 inhibitor 4 (Figure 6), Bondeson et al. designed the RIPK2 PROTAC 12 by conjugating, *via* a PEG linker, a Von Hippel-Lindau (VHL) E3 ligase ligand to the 7-position of the quinoline ring (Figure 15) (Bondeson et al., 2015). 12 was able to degrade RIPK2 in a dose-dependent manner in Human THP-1 monocytes and achieved a 50% protein degradation at a concentration (DC_50_) of 2 nM. The biphasic response was observed for 12, with a bell-shaped dose-response curve indicating that the degradation of RIPK2 was dependent on a PROTAC-mediated ternary complex. Furthermore, the advantage of PROTAC compared to traditional chemotherapy was demonstrated by the sub-stoichiometric catalysis degradation of RIPK2.

In the following study, various E3 ligases have been recruited for RIPK2 degradation by varying the E3 binder of 12, resulting in the design and synthesis of IAP PROTAC 13 and cereblon PROTAC 14 (Figure 15) (Mares et al., 2020).

Among these degraders, IAP-based PROTAC 13 was the most potent compound *in vitro* with DC_50_ = 0.4 nM in THP-1 cells. 12 and 14 were slightly less effective, with DC_50_ of 2.0 nM and 2.5 nM, respectively. As a result of RIPK2 degradation, MDP-induced TNF-α release in a human whole blood assay was greatly inhibited by 13 (IC_50_ = 11 nM). Although 13 was highly active, its liver microsomal (LM) turnover in both rats and humans was very high (11 and 29 mL/min/g LM, respectively). Furthermore, 13 had poor solubility, which required further structure optimization.

Miah et al. optimized the structure of RIPK2 PROTAC 13 by first replacing the RIPK2 binding moiety with the aminopyrazolyl-quinazoline binder (Miah et al., 2021). This change greatly reduced the lipophilicity of the parent compound. With the goal of reducing human microsomal turnover and increasing the potency of RIPK2 PROTACs, the PEG linker was exchanged with a semi-rigid linker constructed from a heteroaromatic ring linked to a piperazine ring and a short alkyl chain. Finally, a new IAP binder was used to improve the stability of the designed PRTOTAC. These modifications resulted in PROTAC 15 as the most suitable degrader for further development in this study (Figure 15). 15 was highly potent in THP-1 cells with RIPK2 DC_50_ = 0.8 nM. Its activity in the critical human whole blood was comparable (IC_50_ = 3.2 nM). 15 displayed a low unbound clearance in rat (217 mL/min/kg). In dog, total systemic clearance of 15 following a 5 min intravenous infusion was low (3.4 mL/min/kg). Even though subcutaneously dosing of 15 demonstrated high levels of RIPK2 degradation and subsequently reduced MDP-stimulated TNFα release, 15 was not orally available. Embedding 15 to a poly (lactic-co-glycolic acid) matrix for slow release partially solves the problem.

## Conclusion

Over the last decade, RIPK2 has emerged as a promising target to modulate immune responses, and a number of drug discovery programs have been launched to identify novel RIPK2 inhibitors and degraders. Repurposing of non-selective kinase (EGFR and MAPK) inhibitors led to preclinical proof-of-concept of inhibition of RIPK2 by small molecules. Clinical development drug candidates (GSK2983559 and BI 706039) have been identified from more comprehensive SBDD programs, which not only increased RIPK2 inhibitor activity but also addressed successfully kinome selectivity, PK and drug safety. Furthermore, RIKP2 degraders (e.g., compound 15) based on the PROTAC approach have demonstrated outstanding efficacy in preclinical models of IBD. The availability of multiple co-crystal structures of RIPK2 in complex with either type I or type II kinase inhibitors enables virtual screen and design of RIPK2 inhibitors with novel chemical scaffolds. Promoting gut tissue repair has emerged as a promising approach for the development of innovative treatments of IBD (Castellanos and Longman, 2019; Chen et al., 2020; Ho et al., 2020). Because of the role of NOD2 in mucosal tissue in IBD patients (Stronati et al., 2008), therapeutic targeting the NOD2/RIPK2 pathway holds potentials to reestablish gut tissue homeostasis (Parlato and Yeretssian, 2014; Rathinam and Chan, 2018). The recent regulatory approvals of JAK inhibitors as oral drugs for the treatment of inflammatory diseases have rekindled enthusiasm in small molecule immunotherapies. Although drug discovery targeting RIPK2 has shown initial promise, broadening the chemical diversity of RIPK2 inhibitors and their mode of action (e.g., orthosteric vs. allosteric) will help mitigate associated risks. In this regard, modulating RIPK2 activity by targeting the CARD-CARD protein-protein interactions between NODs and RIPK2 has not been explored and represents an attractive approach to expand the repertoire in modulating this critical signaling pathway in innate immunity. Because of its broad tissue expression, strategies targeting RIPK2 selectively in inflamed tissue, for instance intestine and colon in IBD, will likely reduce side effects caused by systemic inhibition.
